# Efficacy of modified kidner procedure combined with subtalar arthroereisis treating adolescent type 2 painful accessory navicular with flexible flatfoot

**DOI:** 10.3389/fped.2023.1258032

**Published:** 2023-08-22

**Authors:** Ke Fang, Ting Bi, Ao Hong, Xin Li, Haoli Gong, Bo Li, Fanling Li, Jie Wen, Sheng Xiao

**Affiliations:** ^1^ Department of Pediatric Orthopedics, Hunan Provincial People’s Hospital, The First Affiliated Hospital of Hunan Normal University, Changsha, China; ^2^ Department of Anatomy, Hunan Normal University School of Medicine, Changsha, China

**Keywords:** modified kidner procedure, subtalar arthroereisis, accessory navicular, flexible flatfoot, clinical efficacy

## Abstract

**Purpose:**

To investigate the clinical efficacy of modified kidner procedure combined with subtalar arthroereisis in the treatment of adolescent type II painful accessory navicular with flexible flatfoot.

**Methods:**

From January 2018 to January 2022, 25 adolescent patients (40 feet) with painful type II accessory navicular and flexible flatfoot admitted to our hospital were enrolled in the study, including 13 males (23 feet) and 12 females (17 feet). All patients underwent modified kidner procedure combined with subtalar joint arthrodesis. The Meary's Angle, the first metatarsal Angle of talus (APTMT), the second metatarsal Angle of talus, Pitch Angle, talus tilt Angle, talonavicular coverage Angle (TCA), talus calcaneal Angle (LTCA), and calcaneal Angle were measured on weight-bearing anteroposterior and lateral x-ray films before operation and at last follow-up. The American Orthopaedic Foot and Ankle Society (AOFAS) midfoot score and visual analogue scale (VAS) were used to evaluate the improvement of foot function and pain.

**Results:**

All patients were followed up for average 17.4 ± 2.6 months (12–24). The incisions of 25 patients healed by first intention. The weight-bearing anteroposterior and lateral x-ray films of the foot showed that the suture anchors did not pull out or break, and the foot arch did not collapse further. There was no screw withdrawal or secondary operation to remove the screw in all patients. At the last follow-up, the postoperative visual analogue scale (VAS) score of the affected foot was significantly lower than that before operation (*P* < 0.01), and the American Orthopaedic Foot and Ankle Society (AOFAS) foot function score was significantly higher than that before operation (*P* < 0.01). At the last follow-up, the weight-bearing anteroposterior and lateral foot x-ray films showed that: The Meary's Angle, the first metatarsal Angle of the talus (APTMT), the second metatarsal Angle of the talus, Pitch Angle, talar tilt Angle, talonavicular overbite Angle (TCA), talocalcaneal Angle (LTCA), and calcaneal Angle significantly improved when compared with those before operation (*P* < 0.01).

**Conclusions:**

The modified kidner procedure combined with subtalar arthroereisis has a good clinical effect in the treatment of adolescent type II painful accessory navicular with flexible flatfoot, which can effectively improve the pain symptoms, improve the foot function and imaging manifestations, and correct the flatfoot deformity.

## Introduction

The accessory navicular (AN) bone is a frequently encountered accessory bone, located posteriorly to the navicular bone and often present bilaterally (1). It represents a secondary ossification center of the navicular bone and is inherited as an incomplete autosomal dominant trait, with an incidence rate ranging from 4% to 21% (2–4). Approximately 10% of individuals with AN experience pain symptoms (5). During development, a synchondrosis forms between the accessory navicular and navicular bone ossification centers (4), filled with cartilage and fibrous tissue. Compression, excessive motion, or torsion can trigger painful AN. AN was classified according to Geist (6) into three types: type 1, os tibiale externum, os naviculare secundarium, no cartilaginous connection to the navicular tuberosity. Type 2, prehallux, bifurcate hallux, triangular or heart-shaped, connected to the navicular tuberosity by fibrocartilage or hyaline cartilage. Type 3, an especially prominent navicular tuberosity and is occasionally symptomatic as a result of painful bunion formation over the bony protuberance. Type II AN accounts for approximately 70% of clinically symptomatic AN cases (7). For initial adolescent cases of painful AN, non-surgical treatments such as brace immobilization, anti-inflammatory drug use, and customized corrective insoles are usually preferred. If non-surgical interventions fail to yield results within 3 to 6 months, surgical intervention may be necessary. Surgical options include the Kidner procedure, modified Kidner procedure, simple excision, modified excision, percutaneous drilling, fusion, and modified fusion (8). Among these options, the modified Kidner procedure has advantages as it preserves tendon insertion on other tarsal bones except the AN. Additionally, a suture anchor is inserted into the navicular bone to reinforce the posterior tibial tendon insertion, which promotes early postoperative recovery (9).

The presence of AN can disrupt the normal structure of the posterior tibial tendon (PTT), compromising its ability to support the medial arch of the foot and invert the foot. This can lead to biomechanical alterations in the arch and contribute to the development of flatfoot (10). Recent studies have shown a correlation between AN and flatfoot, with 35% of individuals with AN also exhibiting flatfoot deformities (11). Flatfoot deformities can further contribute to or worsen AN-related symptoms. Treating painful AN alone may not adequately address the underlying flatfoot deformity (12, 13). The Kidner procedure has been shown to alleviate or eliminate AN-related pain. However, its long-term effectiveness in correcting arch collapse remains uncertain (13).

Subtalar arthroereisis is an effective method for the treatment of flexible flatfoot, with good postoperative ankle functions and both radiological and clinical outcomes (14). Biomechanical experiments have demonstrated that the subtalar arthroereisis screw can effectively reduce subluxation between the calcaneus and talus during weight-bearing. This is due to its design, which aligns with the directional characteristics of the sinus tarsi. Implementation of the subtalar arthroereisis screw evenly distributes axial stress across the anterior and posterior aspects of the sinus tarsi (i.e., subtalar anterior and posterior joint surfaces) during weight-bearing (15). This helps restore the normal motion axis of the subtalar joint, correct the force line of the foot, mitigate excessive eversion, and significantly reduce mechanical load on the posterior tibial tendon insertion (16). Currently, there is no standardized treatment for adolescent flexible flatfoot combined with painful AN. Treatment approaches must be tailored based on a comprehensive evaluation of clinical symptoms, imaging findings, and surgical experience. Commonly employed procedures include AN excision and reconstruction of the posterior tibial tendon insertion (17). Therefore, this retrospective study analyzed patients with adolescent type II painful AN syndrome combined with flexible flatfoot who were treated at our hospital between January 2018 and January 2022. The study aimed to investigate the clinical efficacy of a modified Kidner procedure combined with subtalar arthroereisis.

## Methods

Inclusion criteria were as follows: (1) Presence of navicular tuberosity protrusion, tenderness, and meeting the clinical diagnostic criteria for painful AN; (2) Confirmation of type II AN based on foot imaging data; (3) Age between 9 and 14 years; (4) Ineffectiveness of conservative treatment for a minimum of 6 months, including brace fixation, orthotics, oral non-steroidal anti-inflammatory drugs, etc.; (5) Presence of medial arch collapse or disappearance, restoration of the medial longitudinal arch under non-weight-bearing conditions, and meeting the diagnostic criteria for flexible flatfoot: the arch of the foot disappears when the child is standing but re-appears when the child is sitting or standing on tiptoes; and (6) Undergoing a modified Kidner procedure combined with subtalar arthroereisis with at least 1 year of follow-up after surgery.

Exclusion criteria were as follows: (1) Navicular bone fracture; (2) Navicular bone ischemic necrosis; (3) Tarsal fusion; (4) Severe hindfoot valgus deformity (the valgus angle of the hindfoot >15 degree); (5) Type II AN without obvious pain symptoms; (6) History of foot or lower limb surgery on the affected side; (7) Secondary surgery combined with multiple internal medical diseases, neurological or muscular disorders; and (8) Congenital flatfoot or flatfoot caused by iatrogenic factors, trauma, rheumatic diseases, etc.

The study included a total of 25 cases (40 feet), comprising 13 males (23 feet) and 12 females (17 feet), with an age range of 9 to 14 years (mean age 11.28 ± 1.51 years) and a disease duration of 12 to 24 months (mean 17.4 ± 2.6 months). Based on weight-bearing x-ray, CT, and MRI imaging results, all cases were diagnosed with type II AN. The primary reported symptoms were medial foot pain that worsened after exercise. Physical examination revealed significant protrusion at the medial navicular bone of the affected foot, accompanied by local skin redness, swelling, and tenderness upon palpation.

## Surgical procedure

The surgeries were performed by a consistent surgical team. Patients underwent general anesthesia and were placed in a supine position with a pneumatic tourniquet applied to the thigh of the affected limb. A 3–4 cm arc-shaped incision was made centered at the tuberosity of the navicular bone. Layer by layer, the AN and posterior tibial tendon were exposed. The AN was carefully detached, and after cutting its fibrous connection to the navicular bone, it was sharply excised from the tibialis posterior tendon. The remaining end of the posterior tibial tendon insertion was thoroughly cleaned, and any remaining fibrous connections and the navicular bone's tuberosity were repaired using a bone knife and bone forceps. Special care was taken to protect the plantar insertion of the posterior tibial tendon during this process. Subsequently, a suture anchor was placed in the navicular body, and the tibialis posterior tendon insertion was sutured to the fresh bone surface of the navicular bone. The incision was then closed layer by layer, and sterile dressings were applied with pressure, followed by deflation of the pneumatic tourniquet. Then, a 1.5 cm skin incision was made along the skin crease at the sinus tarsi of the affected foot. A minimally invasive scissors was used to carefully separate the sinus tarsi, and the interosseous ligament between the calcaneus and talus was incised. Following the anatomical structure, a guide wire was inserted into the tarsal canal from the anterior lateral to posterior medial direction. Subsequently, the tarsal screw trial was sequentially inserted from small to large along the guide wire, ensuring satisfactory positioning based on anteroposterior, lateral, and calcaneal axial views.

## Postoperative management

The affected limb was elevated and immobilized in an inward rotation long leg plaster cast. After 2 weeks, the sutures were removed. At 6 to 8 weeks, the plaster cast was discontinued, and active toe exercises were encouraged. After 8 weeks, gradual introduction of partial weight-bearing exercises with the aid of custom-made arch supports was initiated. At the 12-month postoperative mark, weight-bearing anteroposterior and lateral x-ray images were taken to assess healing at the osteotomy site, enabling patients to bear full weight and walk normally. At the final follow-up, weight-bearing anteroposterior and lateral x-ray images of the affected foot were used to measure the talar-first metatarsal angle (Meary's angle), anteroposterior talo-first metatarsal angle (APTMT), talar-second metatarsal angle, calcaneal pitch angle (Pitch angle), talar tilt angle, talocalcaneal angle (TCA), lateral talocalcaneal angle (LTCA), and calcaneal angle. The American Orthopaedic Foot and Ankle Society (AOFAS) midfoot score and visual analog scale (VAS) for pain were employed to evaluate treatment outcomes. Occurrence of related complications was recorded.

## Statistical methods

The collected data were analyzed using SPSS 26.0 software. The chi-square test was used for categorical data, and mean ± standard deviation (SD) was used to represent normally distributed data. Intergroup comparisons were conducted using paired *t*-tests. A significance level of 0.05 was applied.

## Results

A total of 25 patients were followed up for 12–24 months after surgery, with an average follow-up duration of 15.2 months. Complete bony union at the fusion site was observed 3 months after surgery. The assessments before surgery and at the last follow-up showed a significant reduction in pain during movement in the AN following surgical treatment. Weight-bearing anteroposterior and lateral x-ray examinations of the affected foot at the last follow-up revealed a significant decrease in the talar-first metatarsal angle (Meary's angle), anteroposterior talo-first metatarsal angle (APTMT), talar-second metatarsal angle, talar tilt angle, talocalcaneal angle (TCA), lateral talocalcaneal angle (LTCA), and calcaneal angle compared to before surgery, with statistical significance (*P* < 0.01). The calcaneal pitch angle (Pitch angle) significantly increased compared to before surgery, with statistical significance (*P* < 0.01). Detailed results can be found in Table 1.

**Table 1:** Comparison of parameters before and after operation.

	Meary's angle	APTMT	Talar-second metatarsal angle	Pitch angle	TCA	Calcaneal angle	Talar tilt angle	LTCA
Pre-OP	15.28 ± 6.03	13.98 ± 6.24	22.45 ± 5.70	13.88 ± 4.33	45.45 ± 6.39	43.28 ± 6.52	33.42 ± 4.60	17.68 ± 2.79
Last FU	3.93 ± 2.09	6.00 ± 4.56	11.78 ± 5.20	20.53 ± 3.27	36.95 ± 7.08	39.40 ± 6.82	21.40 ± 2.05	10.95 ± 3.92
*t*	11.43	8.65	11.32	−11.71	8.451	3.92	15.97	10.16
*P*	<0.01	<0.01	<0.01	<0.01	<0.01	<0.01	<0.01	<0.01

**Figure 1:**
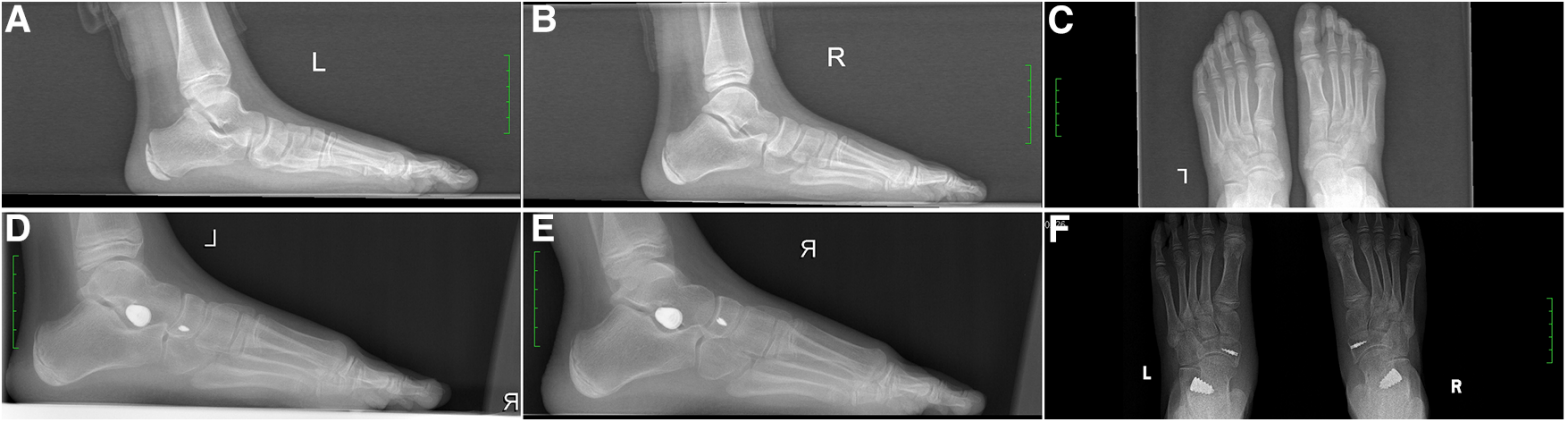
An 11-year-old male patient with bilateral type II painful accessory navicular (AN) combined with flexible flatfoot underwent a modified Kidner surgery combined with subtalar arthroereisis tarsal screw implantation. (**A**) Lateral x-ray images of left feet before surgery: Meary's angle: 16, APTMT: 14, Talar-second metatarsal angle: 19, Pitch angle: 15, TCA: 52, Calcaneal angle: 51, Talar tilt angle: 35, LTCA: 16. (**B**) Lateral x-ray images of right feet before surgery: Meary's angle: 16, APTMT: 11, Talar-second metatarsal angle: 13, Pitch angle: 17, TCA: 51, Calcaneal angle: 51, Talar tilt angle: 33, LTCA: 18. (**C**) AP x-ray images of both feet before surgery, (**D**) Lateral x-ray images of left feet 1-year postoperative follow-up: Meary's angle: 4, APTMT: 3, Talar-second metatarsal angle: 3, Pitch angle: 17, TCA: 36, Calcaneal angle: 49, Talar tilt angle: 24, LTCA: 9. Parameters shows good positioning of the anchor screw and tarsal screw and good alignment of foot arch. (**E**) Lateral x-ray images of right feet 1-year postoperative follow-up: Meary's angle: 4, APTMT: 1, Talar-second metatarsal angle: 3, Pitch angle: 18, TCA: 42, Calcaneal angle: 46, Talar tilt angle: 23, LTCA: 8. Parameters shows good positioning of the anchor screw and tarsal screw and good alignment of foot arch. (**F**) AP x-ray images of both feet 1-year postoperative follow-up, showing correction of AN deformity in both feet.

**Figure 2:**
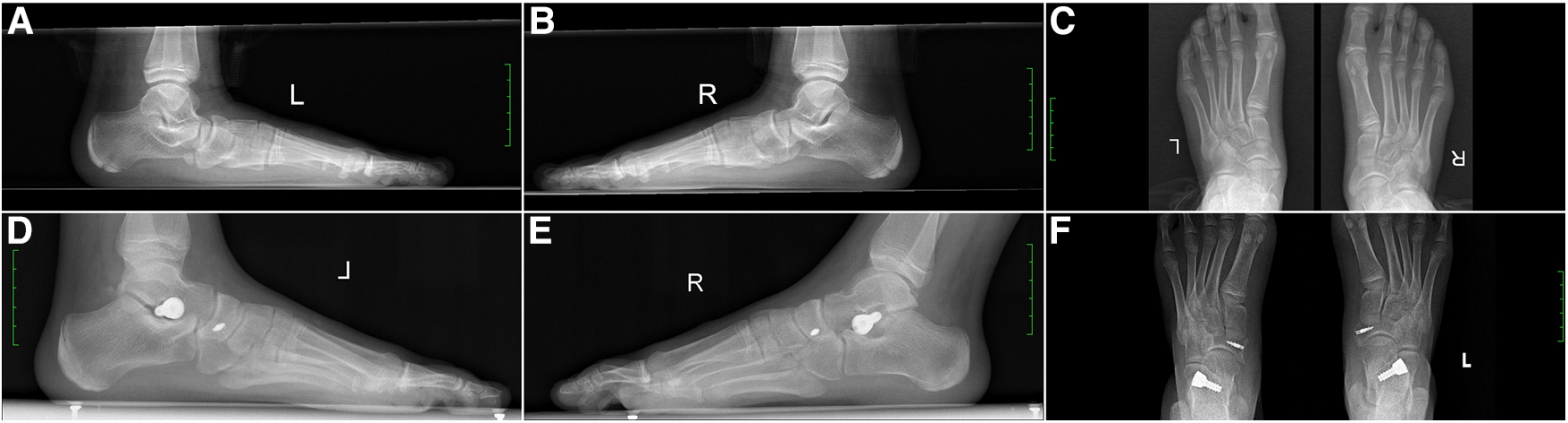
An 10-year-old female patient with bilateral type II painful accessory navicular (AN) combined with flexible flatfoot underwent a modified Kidner surgery combined with subtalar arthroereisis tarsal screw implantation. (**A**) Lateral x-ray images of left feet before surgery: Meary's angle:30, APTMT: 16, Talar-second metatarsal angle: 23, Pitch angle: 16, TCA: 56, Calcaneal angle: 37, Talar tilt angle: 40, LTCA: 20. (**B**) Lateral x-ray images of right feet before surgery: Meary's angle: 20, APTMT: 13, Talar-second metatarsal angle: 20, Pitch angle: 18, TCA: 55, Calcaneal angle: 30, Talar tilt angle: 36, LTCA: 13. (**C**) AP x-ray images of both feet before surgery. (**D**) Lateral x-ray images of left feet 1-year postoperative follow-up: Meary's angle: 5, APTMT: 13, Talar-second metatarsal angle: 10, Pitch angle: 20, TCA: 43, Calcaneal angle: 35, Talar tilt angle: 23, LTCA: 11. Parameters shows good positioning of the anchor screw and tarsal screw and good alignment of foot arch. (**E**) Lateral x-ray images of right feet 1-year postoperative follow-up: Meary's angle: 2, APTMT: 1, Talar-second metatarsal angle: 3, Pitch angle: 14, TCA: 33, Calcaneal angle: 36, Talar tilt angle: 20, LTCA: 5. Parameters shows good positioning of the anchor screw and tarsal screw and good alignment of foot arch. (**F**) AP x-ray images of both feet 1-year postoperative follow-up, showing correction of AN deformity in both feet.

One year after surgery, the visual analog scale (VAS) scores for pain in the affected foot were significantly lower compared to the preoperative scores in the same group, with statistical significance (*t* = 14.50, *P* < 0.01). Furthermore, there was a significant improvement in the American Orthopedic Foot and Ankle Society (AOFAS) foot function scores of patients compared to their preoperative scores, with statistical significance (*t* = −17.98, *P* < 0.01). The results are presented in Table 2. Typical cases are illustrated in Figures 1, 2.

**Table 2:** Comparison of AOFAS and VAS before and after operation.

	*n*	Pre-OP	Last FU	*t*	*P*
AOFAS	25	47.40 ± 6.98	82.84 ± 6.09	−17.98	<0.01
VAS	25	5.28 ± 0.98	1.72 ± 0.84	14.50	<0.01

All patients experienced primary wound healing without any instances of infection, hematoma, nerve injury, or calcaneal anterior process fracture. There were no reported cases of subtalar arthroereisis screw loosening or the need for a second surgery to remove the screw. One case (1 foot) experienced pain in the tarsal sinus area, which was considered related to the slightly larger size of the tarsal screw. The symptom resolved with a single regional anesthetic injection. Two cases (2 feet) had mild residual AN pain after surgery, which was attributed to insufficient correction of hindfoot valgus. The symptoms of both feet improved after using custom foot pads for one year. Anteroposterior and lateral x-ray examinations of the affected foot revealed that the suture anchors had not loosened or broken, and there was no further collapse of the foot arch.

## Discussion

The accessory navicular bone is a common variation in the skeletal structure of the foot and is one of the most frequent symptomatic accessory bones. Its exact cause is still unclear, but it has been recognized for a long time, first described by Bauhn in 1605 (18). While the presence of the accessory navicular bone usually does not cause symptoms, it can lead to painful AN when accompanied by medial foot pain. Painful AN is associated with factors such as local irritation of the accessory navicular prominence, abnormal traction of the Achilles tendon, and pathological changes in the accessory navicular itself (19). Patients typically experience pain, tenderness, swelling, and a palpable prominence along the medial arch between the ages of 11 and 15, with symptoms worsening during weight-bearing, walking, physical activity, and wearing shoes. The presence of the accessory navicular bone alters the shape and insertion point of the Achilles tendon, affecting its function and leading to flat feet and strain. The Achilles tendon plays a crucial role in maintaining the medial longitudinal arch and its dysfunction can result in arch collapse and characteristic secondary deformities of flat feet (20, 21). Studies have shown that 35% of patients with accessory navicular exhibit flat foot deformities (11). These deformities are characterized by the absence of the medial longitudinal arch, forefoot abduction, hindfoot valgus, and often accompanied by Achilles tendon contracture.

Patients with painful accessory navicular (AN) in the foot commonly present with pain and tenderness on the medial side of the midfoot. Physical examination often reveals swelling and tenderness specifically on the medial side of the navicular bone. For patients with painful AN and flexible flat feet, conservative treatment is generally recommended. This may involve wearing corrective shoes, using braces, receiving massages, and undergoing muscle training (22). If symptoms persist despite conservative measures, surgical treatment may be considered as an option (17). The goal of surgical treatment for painful AN is to improve symptoms, restore the function of the posterior tibial tendon, improve the force line, and reshape the arch of the foot. The Kidner procedure, first proposed in 1929, is considered the standard surgical procedure for treating painful AN. However, the traditional procedure has drawbacks such as significant trauma, slow recovery, and the need for long-term immobilization. To address these limitations, the modified Kidner procedure has been developed. This procedure not only retains the advantages of the traditional procedure but also preserves the tendon's insertion point on other tarsal bones besides the accessory navicular (AN). Additionally, a wire anchor is inserted into the navicular bone to reinforce the tendon's insertion point, promoting early postoperative recovery. Several studies have reported successful outcomes with the modified Kidner procedure. Lee et al. (23) achieved a clinical better result by performing modified Kidner procedurein 14 cases of type II painful AN. Kim et al. (24) utilized the modified Kidner procedure in patients with painful AN, with 7 cases showing excellent results and 4 cases showing good results. The procedure is considered simple to perform, provides strong grip strength, and has minimal impact on the navicular bone. These factors contribute to effective tightening of the posterior tibial tendon and strengthening of its insertion point on the navicular bone, facilitating early postoperative recovery. However, our team's findings suggest that the improvement achieved with a single Kidner procedure in improving the foot arch is relatively limited compared to previous reports (25). In Garras's study (11), three patients required removal of the arthroereisis plug because of impingement pain, but in our cases, we had no instances of arthroereisis screw loosening and no need for additional surgery to remove the screw. Garras suggests that the pain may be caused by larger screws. But as our patients are younger than those of Garras, they may have more growth potential and tolerate the screws better. Specifically, we observed slight improvements in the Pitch angle and TMA ap, measuring only 1.9° and 0.7°, respectively, compared to preoperative values (26). Previous research has also indicated that regardless of whether Kidner surgery or AN fusion is performed, the calcaneal pitch angle increases but does not significantly affect the TNCA (Talo-navicular coverage angle) and T1MT (Talo-first metatarsal angle) (27, 28). Biomechanical experiments have demonstrated the effectiveness of the subtalar arthroereisis screw in reducing subluxation between the talus and calcaneus during weight-bearing. Its design aligns with the orientation of the sinus tarsi, enabling even distribution of axial stress across the anterior and posterior aspects of the sinus tarsi, which are the anterior and posterior joint surfaces of the subtalar joint (15). Consequently, the subtalar arthroereisis screw restores the normal motion axis of the subtalar joint, corrects the force line of the hindfoot, restores the foot arch, eliminates excessive valgus, and significantly reduces the mechanical load on the posterior tibial tendon insertion point. Therefore, the subtalar arthroereisis screw has the potential to alleviate pain and correct the deformity associated with flat feet (29). Ruiz Picazo et al. (30) have also reported the effectiveness of the subtalar arthroereisis screw in treating flexible flat feet, leading to good postoperative functionality and imaging results. This serves as one of the theoretical foundations for this clinical study. In recent years, the use of the subtalar arthroereisis screw has gained popularity as a safe, effective, and minimally invasive treatment option, demonstrating significant improvement in patients with flat foot deformity (31, 32).

This study investigated the use of the modified Kidner procedure combined with the subtalar arthroereisis screw in 25 cases (40 feet) of adolescent patients with painful accessory navicular (AN) and flexible flat feet. The aim was to prevent the development of later varus deformity of the high arch through early intervention. The imaging results demonstrated significant reductions in various angles, including the talar-first metatarsal angle (Meary's angle), anteroposterior talo-first metatarsal angle (APTMT), talar-second metatarsal angle, talar tilt angle, talocalcaneal angle (TCA), lateral talocalcaneal angle (LTCA), and calcaneal angle compared to the pre-surgery measurements. These reductions were statistically significant (*P* < 0.01). Additionally, the calcaneal pitch angle (Pitch angle) significantly increased compared to the pre-surgery measurements, also with statistical significance (*P* < 0.01). All patients achieved successful wound healing without any complications such as wire anchor or tarsal screw loosening, detachment, or removal. The modified Kidner procedure combined with the subtalar arthroereisis screw yielded favorable outcomes in terms of AOFAS foot and VAS scores, with statistically significant differences observed before and after surgery. This treatment not only alleviated preoperative pain symptoms at the navicular bone but also resulted in better correction of the flat foot deformity. Moreover, it facilitated a faster recovery of the foot arch and reduced the duration of non-weight-bearing requirements. Throughout the study, no cases required the removal of the wire anchor or tarsal screw. It is believed that the tarsal screw, which involves solely soft tissue surgery, does not impact the foot development of adolescents. In cases without symptoms, the removal of the tarsal screw was not recommended. However, individual variations among patients may affect the optimal customization of tarsal screws, potentially leading to mild overcorrection and post-surgical complications such as pain. Future research could explore the use of 3D printing technology for personalized customization of tarsal screws.

This study has some limitations. This was a retrospective study with a small sample size which may lead to bias. Future biomechanical studies of subtalar arthroereisis in children would be useful.

In conclusion, the combination of the modified Kidner procedure and tarsal screw insertion represents a departure from the traditional approach of using a single procedure. The authors argue that the treatment of adolescent patients with painful AN and flexible flat feet should encompass both interventions. The key focus of treatment is to stabilize the subtalar joint, remove the accessory navicular bone, reconstruct the posterior tibial tendon, and alleviate pain. This comprehensive approach effectively restores the normal biomechanical structure of the foot and ankle, improves foot function and imaging results, and corrects the deformity associated with flat feet.

## Data Availability

The raw data supporting the conclusions of this article will be made available by the authors, without undue reservation.
